# Point-Of-Care Ultrasound to Diagnose Molar Pregnancy in the Emergency Department: A Case Report and Topic Review

**DOI:** 10.7759/cureus.68223

**Published:** 2024-08-30

**Authors:** Erin Avers, David M Langley, Katelyn Karalic, Ryan Snitowsky

**Affiliations:** 1 Emergency Medicine, Lake Erie College of Osteopathic Medicine, Bradenton, USA; 2 Emergency Medicine, Florida State University College of Medicine, Sarasota, USA

**Keywords:** hydatidiform mole, obstetrical emergencies, first-trimester of pregnancy, gestational trophoblastic disease (gtd), molar pregnancy, pocus in emergency medicine

## Abstract

Molar pregnancy is a topic in emergency medicine frequently tested and regularly discussed but is perhaps overshadowed by other conditions such as ectopic pregnancy. It is a rare diagnosis encountered in the emergency department (ED) and is part of a broad spectrum of pathological conditions that fall into the category of gestational trophoblastic disease (GTD). Diagnosis of this potentially malignant condition requires the emergency physician to bear this condition in mind when treating any woman while considering obstetric-related conditions in the first trimester, vaginal bleeding, pelvic pain or pressure, and excessive nausea and vomiting. We present the case of a 20-year-old primigravida 12-week pregnant female who presented to the ED sent in by the midwifery clinic for evaluation with concerns for absent fetal heart tones and abnormal uterine appearance. Point-of-care ultrasound (POCUS) performed upon arrival demonstrated an irregular complex echogenic uterine mass with anechoic areas and cystic structures suspicious for a molar pregnancy. Obstetrics-gynecology (OB-GYN) admitted the patient promptly for definitive surgical care, and tissue analysis confirmed a complete molar gestation. This case highlights the effectiveness of POCUS for prompt diagnosis and treatment of molar pregnancy in the ED.

## Introduction

Gestational trophoblastic disease (GTD) is a group of rare diseases characterized by the growth of abnormal trophoblastic cells inside the uterus after conception [1]. The most common form of GTD is a hydatidiform mole, or molar pregnancy, a non-viable pregnancy where trophoblastic proliferation arises from the placenta. Moles are either complete (46 XX or XY without fetal tissue) or partial (69 XXX or XXY with fetal tissue present) [2]. The pathogenesis involves an abnormal conception with two copies of the paternal genome with an absent (complete mole) or present (partial mole) maternal genome. Hydatidiform moles are benign but have the potential for malignant transformation, with complete moles carrying higher risk (15-20%) than partial (0.5-1%) [3]. Extremes of reproductive age and history of prior GTD are risk factors for molar pregnancy, and the incidence of hydatidiform mole is 1-2 per 1000 pregnancies [4].

The most aggressive form of GTD and the most worrisome outcome of untreated hydatidiform mole is choriocarcinoma, with 30% of cases already metastasized at the time of diagnosis. The incidence is one per 40,000 pregnancies and the risk of developing choriocarcinoma after a complete molar pregnancy is 2-3% [2]. Clinically molar pregnancies typically present with irregular vaginal bleeding during the first trimester. Sonography is an excellent diagnostic tool for visualizing tumors, with a classic “snowstorm” appearance characteristic for complete hydatidiform moles [5]. Histological examination is the gold standard for confirmation of the disease. In complete moles, beta-human chorionic gonadotropin (beta-hCG) levels are typically inappropriately high and reach concentrations greater than 100,000 IU/L [6]. 

The average gestational age at diagnosis is 9 weeks, with earlier diagnosis correlating with less risk for anemia, hyperemesis, pre-eclampsia, or hyperthyroidism. Molar pregnancies are treated with uterine evacuation and histological examination of the products. The patient is followed with serum or urine beta-hCG levels until the value returns to normal. Surveillance is warranted with beta-hCG as a biomarker of treatment failure, disease recurrence, or malignant transformation [6].

## Case presentation

A 20-year-old female gravida 1 para 0 at 12 weeks gestation presented to the emergency department (ED) after a prenatal ultrasound at a midwifery clinic revealed absent fetal heart tones and abnormal uterine appearance. She was instructed to follow up with obstetrics-gynecology (OB-GYN) because her midwife could not manage the pregnancy. Having been turned away by several offices when attempting to make an appointment, she was prompted to go to the ED. Since becoming pregnant, she had daily nausea and vomiting and a seven-pound weight loss for which she visited an outside ED at 8 weeks gestation and was provided ondansetron with some relief of her symptoms. She denied abdominal pain or vaginal bleeding.

**Figure 1:**
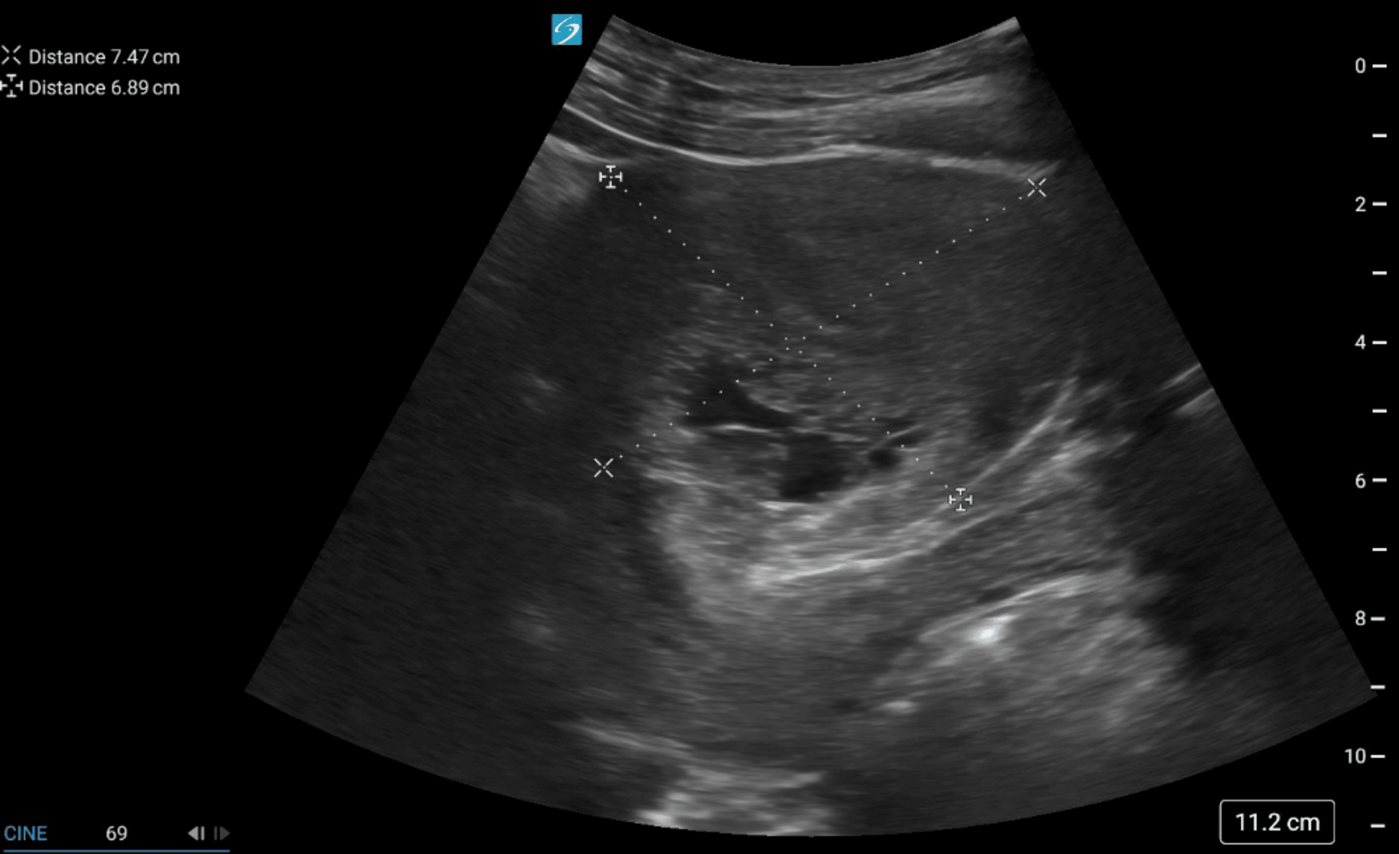
POCUS revealing an irregular complex echogenic mass within the uterus and anechoic areas with cystic structures POCUS: Point-of-care ultrasound

On exam, the patient had a blood pressure of 97/60, while the remaining vital signs were normal. Pelvic examination revealed the uterus was palpable just above the pubic symphysis and minimally tender to palpation and there were no palpable adnexal masses. A point-of-care ultrasound (POCUS) was performed and no visualized intrauterine pregnancy (IUP) was seen. It revealed an irregular complex echogenic mass within the uterus with anechoic areas and cystic structures (Figure 1).

**Figure 2:**
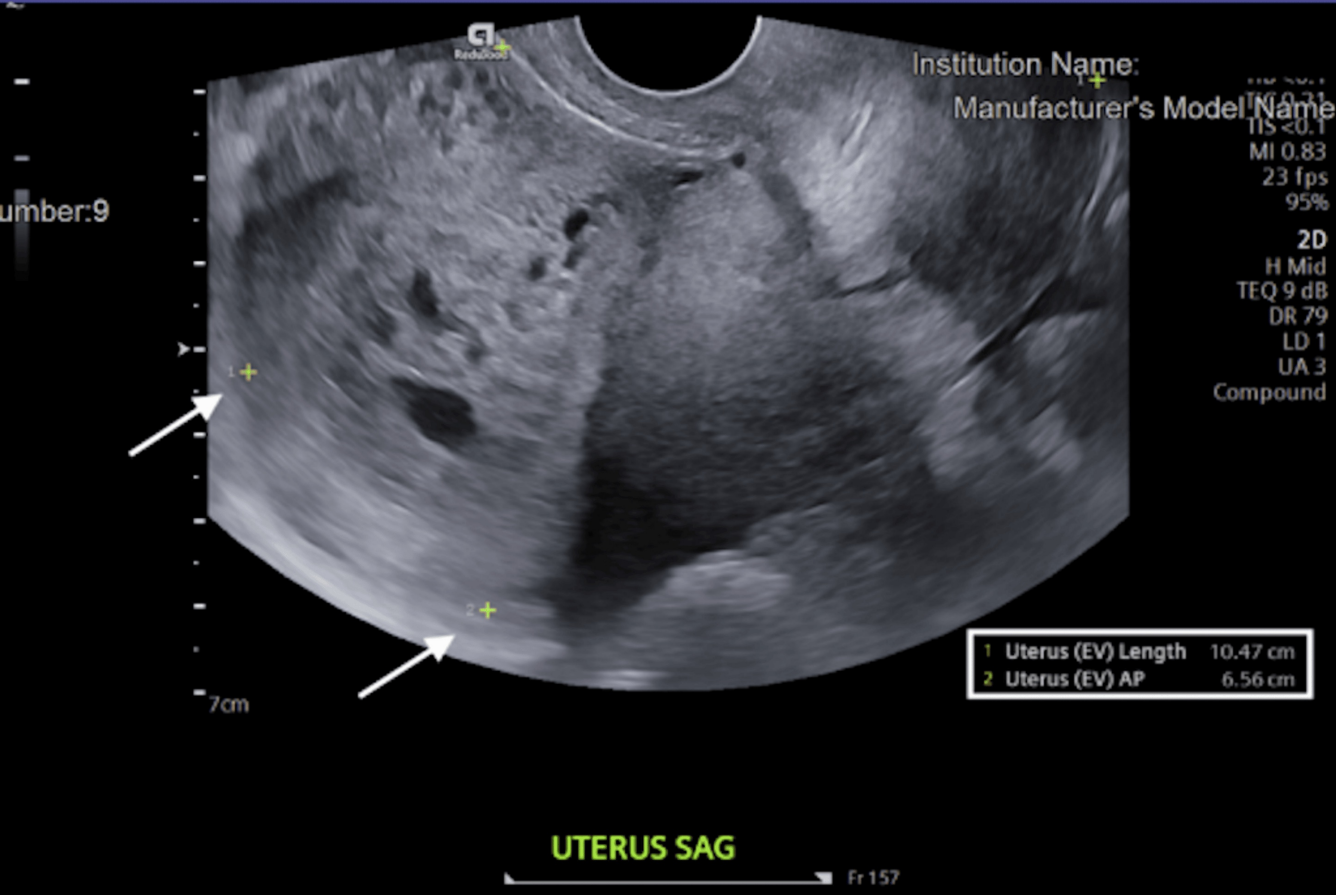
TVUS showing distortion and thickening of the endometrium with grossly abnormal heterogeneous and cystic components with suggested invasion into the myometrium TVUS: Transvaginal ultrasound

This “snowstorm-like” appearance raised concern for molar pregnancy, prompting immediate OB-GYN consultation. The serum beta-hCG level was measured at 56,857 mIU/mL. See Table 1 for reference ranges. An additional radiology-performed ultrasound was obtained and revealed the uterine size to be 10.5 x 6.6 x 8.3 cm with distortion, thickening of the endometrium, and grossly abnormal heterogeneous and cystic components with suggested invasion into the myometrium. There was also a 1.5 x 1.4 x 1.6 cm right corpus luteal cyst. The left ovary was not visualized (Figure 2).

**Table 1:** Laboratory reference range values for beta-hCG based on estimated gestational age mIU/mL: Milli-international units per milliliter

Reference Values		Patient’s Values
Gestational age	beta-hCG (mIU/mL)	beta-hCG (mIU/mL)
Negative	<3	-
2 - 3 months	10,000 - 100,000	56,857

**Figure 3:**
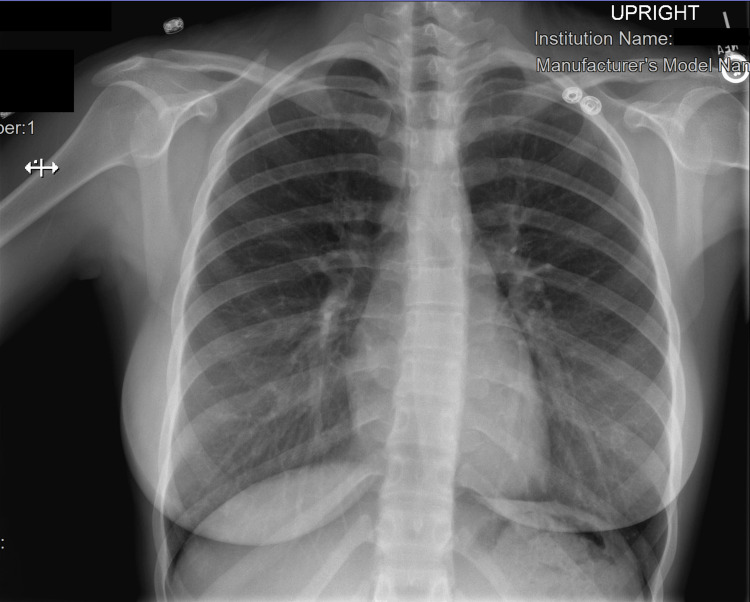
Chest x-ray of the patient showing mild levoscoliosis but no acute cardiopulmonary processes

OB-GYN admitted the patient for a presumed molar pregnancy and scheduled her for surgical evacuation of the mass. She underwent ultrasound-guided dilation and curettage with suction the following day which she tolerated well. The pathology report confirmed a complete molar pregnancy. Before discharge, a chest-x ray was obtained to evaluate for lung lesions, which showed mild levoscoliosis but no acute cardiopulmonary disease (Figure 3); a beta-hCG was repeated on hospital day 1 and was 38,070 mIU/mL. At follow-up one week later her beta-hCG continued to trend down to 2642 mIU/mL and at the two-week follow-up appointment dropped to 359 mIU/mL. She stated that she was feeling well and was scheduled for a follow-up to ensure further downtrending of the beta-hCG levels.

## Discussion

This case brings an opportunity to discuss multiple teaching points from an ED perspective. First, we would like to emphasize how POCUS can be used as a diagnostic tool not only for molar pregnancies but also for many obstetrics-related complaints. For pregnant patients in the ED, ultrasound is a quick and effective way to assess for IUP. It cannot be overstated that ectopic pregnancy is the most important consideration until it can be either confirmed or excluded with conviction. POCUS can effectively assess other life-threatening obstetric complications, including ectopic pregnancy demonstrations, with a sensitivity as high as 99.3% [7].

Pelvic ultrasound and beta-hCG should be used together to evaluate for IUP and its complications [8]. For transabdominal ultrasound (TAUS), an IUP should be detectable when the beta-hCG level reaches about 6000 mIU/mL [8,9] and for transvaginal ultrasound (TVUS) some studies have shown 99% sensitivity detection of IUP when the level is over 3510 mIU/L [10]. Despite the earlier detection of IUP by TVUS, between TVUS and TAUS, TAUS is by far more frequently performed in the ED by ED physicians as one survey showed that as few as 20% of ED physicians use TVUS regularly [11]. As several authors have shown, in molar pregnancies the beta-hCG is much higher than expected, with levels above 100,000 mlU/mL [4,8,9]. If a patient presents with higher-than-expected levels and ultrasound shows a “snowstorm appearance” of the uterine cavity, molar pregnancy can be suspected and urgent obstetrics consultation should be obtained [4,8,9].

Hydatidiform moles are rare and typically occur at extremes of reproductive age, with the most common presenting symptom being first-trimester vaginal bleeding. The patient is of average reproductive age and had no vaginal bleeding, emphasizing the point that the threshold for ultrasound should be low in patients with pregnancy-related concerns. At her prior ED visit for hyperemesis, ultrasound may have been able to aid in earlier diagnosis and treatment of a disease that has the potential to transform into a metastatic and life-threatening disease.

Additionally, the possibility of molar pregnancy should be considered if there is disproportionate nausea and vomiting, severe anemia, heavy vaginal bleeding, hyperthyroidism symptoms, hypertension before 24 weeks, abnormally high beta-hCG levels, uterine size larger or smaller than expected, or there are ultrasound findings suggestive of the diagnosis [11]. Furthermore, some have suggested that beta-hCG levels be evaluated in reproductive-age women, with any abnormal bleeding or symptoms that could be caused by a malignancy. It is to facilitate early diagnosis and treatment of GTD. Further testing, including pelvic ultrasound, should be considered in addition to beta-hCG [12]. In the ED, the threshold should be low to obtain POCUS imaging to evaluate for IUP associated with a decreased length of ED stay [13,14]. To further strengthen this argument, some moles may not produce beta-hCG at all, thus leading the clinician to rely on other diagnostic tools [15].

The patient was 12 weeks gestation at the time of diagnosis, which is three weeks later than the average time (9 weeks) of diagnosing molar pregnancies. If a patient is seeking care where POCUS is not available at that time, quantitative beta-hCG may be able to prompt the clinician to consider GTD when levels are higher than expected for gestational age. While midwives are indispensable in providing care during pregnancy, we suggest that patients who visit the ED for pregnancy complications follow up with a board-certified OB-GYN.

## Conclusions

GTD is an important consideration for the emergency medicine physician. If suspected, an OB-GYN consultation should be obtained urgently. It must be stated again that ectopic pregnancy is the most important consideration and is on the differential diagnosis until it can be either confirmed or excluded with conviction. While hyperemesis was the presenting symptom for the patient, we want to emphasize the spectrum of presentations for molar pregnancy and suggest that patients visiting the ED for complications of pregnancy should have a low threshold for POCUS to evaluate for IUP.
